# Identifying National Availability of Abortion Care and Distance From Major US Cities: Systematic Online Search

**DOI:** 10.2196/jmir.9717

**Published:** 2018-05-14

**Authors:** Alice F Cartwright, Mihiri Karunaratne, Jill Barr-Walker, Nicole E Johns, Ushma D Upadhyay

**Affiliations:** ^1^ Advancing New Standards in Reproductive Health Department of Obstetrics, Gynecology, and Reproductive Sciences University of California, San Francisco Oakland, CA United States; ^2^ University of California, Berkeley Berkeley, CA United States; ^3^ Zuckerberg San Francisco General Library University of California, San Francisco San Francisco, CA United States

**Keywords:** abortion seekers, reproductive health, internet, access to information, information seeking, abortion patients, reproductive health services, information seeking behavior

## Abstract

**Background:**

Abortion is a common medical procedure, yet its availability has become more limited across the United States over the past decade. Women who do not know where to go for abortion care may use the internet to find abortion facility information, and there appears to be more online searches for abortion in states with more restrictive abortion laws. While previous studies have examined the distances women must travel to reach an abortion provider, to our knowledge no studies have used a systematic online search to document the geographic locations and services of abortion facilities.

**Objective:**

The objective of our study was to describe abortion facilities and services available in the United States from the perspective of a potential patient searching online and to identify US cities where people must travel the farthest to obtain abortion care.

**Methods:**

In early 2017, we conducted a systematic online search for abortion facilities in every state and the largest cities in each state. We recorded facility locations, types of abortion services available, and facility gestational limits. We then summarized the frequencies by region and state. If the online information was incomplete or unclear, we called the facility using a mystery shopper method, which simulates the perspective of patients calling for services. We also calculated distance to the closest abortion facility from all US cities with populations of 50,000 or more.

**Results:**

We identified 780 facilities through our online search, with the fewest in the Midwest and South. Over 30% (236/780, 30.3%) of all facilities advertised the provision of medication abortion services only; this proportion was close to 40% in the Northeast (89/233, 38.2%) and West (104/262, 39.7%). The lowest gestational limit at which services were provided was 12 weeks in Wyoming; the highest was 28 weeks in New Mexico. People in 27 US cities must travel over 100 miles (160 km) to reach an abortion facility; the state with the largest number of such cities is Texas (n=10).

**Conclusions:**

Online searches can provide detailed information about the location of abortion facilities and the types of services they provide. However, these facilities are not evenly distributed geographically, and many large US cities do not have an abortion facility. Long distances can push women to seek abortion in later gestations when care is even more limited.

## Introduction

Women’s ability to determine if and when they get pregnant and continue that pregnancy is key to their overall well-being. Women who are denied wanted abortions experience some negative outcomes compared with women who were able to obtain abortions, including increased economic insecurity [1] and continued exposure to violence from the man involved in the pregnancy [2]. While abortion rates have declined slightly in recent years, over 926,000 abortions were performed in the United States in 2014 [3]. This rate is equivalent to 1 in 4 women of reproductive age having an abortion within her lifetime [3], which underscores that abortion is common.

The explanations for the decline in abortion rates are varied, but part of this drop can likely be attributed to the decrease in facilities at which women can obtain abortion care across the United States over the past decade. Most abortions (95%) are performed in specialized abortion clinics (rather than private physicians’ offices or hospitals), and the number of these clinics declined in half of US states from 2011 to 2014, with some regions experiencing up to a 22% decrease [3]. Because 90% of US counties do not have an abortion provider [3], many women seeking abortion must travel outside their home counties to obtain care. Other geographic disparities have been documented: women living in rural areas, the South and Midwest regions of the United States, and those seeking second-trimester or later abortions are more likely to travel farther for services, often 50 miles (80 km) or more one way [4-7]. These shifts in the availability of abortion-providing facilities indicate that women in underserved areas must travel increasingly far for abortion care.

Somedecline in the number of abortion facilities may be due to the more than 400 state laws regulating abortion that have been adopted between 2011 and 2017 [8], which, among other requirements, mandate that physicians have local hospital admitting privileges, facilities have formal transfer agreements with local hospitals, and facilities become ambulatory surgical centers. These laws have likely led to the closure of facilities that could not meet the financial or administrative requirements imposed by these laws. For example, after these types of laws were passed in Texas in 2013, the number of abortion facilities decreased by 54% over 15 months, requiring women whose nearest clinic had closed to travel 85 miles (137 km) one way to a facility [9]. Additional analyses of trends in abortion rates in Texas from 2012 to 2014 found a relationship between increases in distance to the nearest abortion facility and decreases in the county abortion rate [10]. Another analysis from Louisiana estimated that, if admitting privileges laws were to go into effect, 67% of women of reproductive age would live more than 150 miles (241 km) from the nearest abortion facility, thereby tripling the distance women have to travel to reach the nearest facility for care [11,12]. With distance come increased travel time, increased costs for transportation and childcare, lost wages, the need to take time off of work or school, the need to disclose the abortion to more people than desired, and overall delays in care [13-15]. Ultimately, delays in reaching and obtaining care can push women later into their pregnancies, even up to the point that they might not be able to obtain a wanted abortion, depending on the gestational limits on abortion in their state [16].

To obtain abortion care in their communities, women who do not know where to go may use the internet to find abortion facility information [15]. Almost half (45%) of women seeking abortion services at clinics in Nebraska located the abortion clinic through an online search [17], and a recent study documented an interest in information on self-abortion among people searching online using the search engine Google [18]. Online searching for abortion information appears to be more prevalent in states with restrictive abortion laws and where abortion availability is limited, suggesting that women with reduced access to abortion are more likely to seek out information on abortion online [19,20].

We were interested in examining the question “What does the current landscape of abortion facilities look like to women searching online for abortion services?” There are no publicly available systematically documented and comprehensive lists of US abortion facilities, which makes it difficult to determine how far women must travel to obtain these services. Considering the trends in increased restrictions and decreasing numbers of abortion-providing facilities, it is important to generate accurate estimates of the distances women must travel to obtain abortion services in order to demonstrate potential impacts of closures. This study aimed to address this question by documenting the location of and abortion services available at abortion facilities identified through a systematic online search in all 50 US states (and the District of Columbia) and then calculating travel distances to these facilities from metropolitan areas with populations of 50,000 or more.

## Methods

### Data Collection

We conducted a systematic online search for abortion facilities using the Google (Google LLC), Bing (Microsoft Corporation), and Yahoo (Oath Inc) search engines between February 22, 2017 and May 22, 2017. Although Google alone accounts for a substantial portion of the market share in the United States (87.5%), together the 3 search engines comprised 99.1% of the total search engine market share as of February 2017 [21]. We conducted a search with the keywords “Abortion clinic in [state]” (no quotes) for all 50 states and the District of Columbia in each of the 3 search engines. In addition, we searched all cities (n=302) with populations over 100,000 based on 2015 US Census population estimates [22] using the keywords “Abortion clinic in [city]” (no quotes). For states that had fewer than 3 cities with populations over 100,000, we used the 3 most populous cities from the same US Census source. We conducted the keyword searches in Google’s Chrome browser on Incognito mode and cleared the complete browsing history, including cookies and other site data and cached images and files, prior to each search. The researcher was logged into a Google account created specifically for this study during the searches. We chose keyword searches to reflect the natural language that women would use to search for local abortion services.

We assessed the first 20 results for each *city* for information on abortion-providing facilities, similar to previous analyses of search engine content [23,24], resulting in a review of a total of 18,120 city results across all 3 search engines. To capture the larger number of facilities expected in statewide searches, we reviewed the first 30 results for each state (for a total review of 4590 state results). For each result, we examined the website for relevant information. If the website belonged to an abortion facility, we included the result in our count of facilities and recorded whether they provided medication abortion, or aspiration or surgical abortion, as well as the facility gestational limit. Some facilities noted on their websites that they offered services beyond the gestational limit on a case-by-case basis; however, we recorded the limit that each facility offered to all patients seeking services. We included hospitals and clinics associated with universities and medical schools through the Ryan Residency Training Program in Abortion and Family Planning [25] in the analysis if they provided information about availability of abortion services on their website, even if they did not come up in our systematic Web searches. If a website did not provide information about a facility where abortion care could be obtained or explicitly stated that they did not provide abortion care, we excluded the facility. If the online information was incomplete or unclear, we called the facility using a mystery shopper method, which simulates the perspective of patients calling for services [26]. With these calls, we verified that it was not a crisis pregnancy center, confirmed that the facility was open and providing abortion services, and obtained additional information on its address, including state and zip code, types of abortion services provided, and gestational limits. Finally, because Planned Parenthood is the health care provider most widely known to provide abortion services in the United States, we reviewed all facilities listed by state on the Planned Parenthood Federation of America website as a validity check against the results from our systematic search. We confirmed that all Planned Parenthood facilities providing abortion had been captured by our searches. The study was approved by the institutional review board of the University of California, San Francisco.

### Data Analysis

We described the number of facilities and the proportion of facilities that offered medication abortion only, aspiration or surgical abortion only, and both medication and aspiration or surgical abortion in each state and region. We grouped states by region and subregion based on US Census categories. The latest gestational limit at which facilities offered aspiration or surgical abortions was documented for each state. Using 2015 population estimates taken from 2010 US Census data [22], we determined the number of women of reproductive age (15-49 years) per abortion facility in each state.

To calculate the cities farthest from an abortion-providing facility, we defined cities based on the US Census’s data on incorporated places of 50,000 or more [22], which amounted to 758 cities. After removing those cities that had at least one abortion provider, we calculated the distance from each city to all the abortion facilities within the state and in any neighboring or nearby states. For each city, we then took the minimum of these distances to determine the closest provider. We calculated distances in Stata 14 (StataCorp LLC) using the traveltime3 command, which uses a Google Maps application programming interface to calculate driving distances in miles and time. Mapping was performed in Redivis, a Stanford University-based online visualization platform. Rather than using Euclidean (straight-line) distance, Redivis uses road network information, including road type and corresponding average speed, sourced from OpenStreetMap [27] to implement a cost-distance algorithm to predict distance-access to abortion facilities.

## Results

### Distribution and Characteristics of Abortion Facilities in the United States

We identified 780 abortion facilities in the United States. The distribution of abortion facilities was not uniform across states. The largest numbers of facilities were in the Northeast and the West. California had the highest number of facilities (n=152), while Kentucky, Mississippi, Missouri, North Dakota, South Dakota, and West Virginia had 1 facility each. The geographic region with the highest ratio of women of reproductive age to facility was the Midwest, with 165,886 women per abortion facility (Table 1). The Northeast had the lowest ratio (55,662:1). While population density is not distributed evenly across all regions, the subregions with the highest ratios were the West South Central and East South Central subregions, with 298,733 and 288,463 women per facility, respectively. The state with the highest ratio of women to facility was Missouri, with 1,365,575 women per facility, and the lowest was in Maine, with 13,905 women per facility.

Most facilities reported providing both medication and aspiration or surgical abortion, although the proportion of facilities that provided different types of abortion care also varied by region and state. Over 30% (236/780, 30.3%) of all the facilities reported on their websites that they only provided medication abortion (Table 2), while 65.4% (510/780) provided both medication and aspiration or surgical abortion. Very few offered just aspiration or surgical abortion.

The South region had the highest proportion of facilities offering both medication abortion and aspiration or surgical abortion (169/193, 87.6%). While the Northeast and West had many more facilities overall, almost 40% of facilities in each of these regions offered medication abortion only (89/233, 38.2%; and 104/262, 39.7%, respectively). When looking at subregion, New England (34/74, 45.9%) and Pacific (87/207, 42.0%) had even greater proportions of facilities offering medication abortion only.

The highest gestational limit advertised by facilities also varied by state, subregion, and region (Table 2). The states with the lowest advertised gestation for abortions were Wyoming (12 weeks) and Indiana and South Dakota (both 13 weeks and 6 days), and the lowest subregions were West North Central in the Midwest and East South Central in the South, with limits of 22 weeks. Among all facilities, 50.9% (397/780) provided abortion services at 14 weeks or later and 26.5% (207/780) provided services at 20 weeks or later.

**Table 1:** Number of US abortion facilities by region and state, May 2017.

Region and state			Number of facilities	Population of women of reproductive age (ages 15-49 years) per facility
United States			780	95,033
**Northeast**			233	55,662
	**New England**		74	45,655
		Connecticut	20	40,632
		Maine	20	13,905
		Massachusetts	19	84,973
		New Hampshire	6	48,395
		Rhode Island	3	82,130
		Vermont	6	22,743
	**Middle Atlantic**		159	60,320
		New Jersey	50	40,927
		New York	92	51,293
		Pennsylvania	17	166,210
**Midwest**			92	165,886
	**East North Central**		68	155,508
		Indiana	6	250,997
		Illinois	25	120,135
		Michigan	23	96,054
		Ohio	11	235,016
		Wisconsin	3	423,591
	**West North Central**		24	195,289
		Iowa	9	75,629
		Kansas	4	161,029
		Minnesota	5	245,486
		Missouri	1	1,365,575
		Nebraska	3	140,140
		North Dakota	1	167,601
		South Dakota	1	181,145
**South**			193	145,645
	**South Atlantic**		147	98,787
		Delaware	3	70,851
		District of Columbia	3	66,863
		Florida	65	67,757
		Georgia	17	145,646
		Maryland	25	56,665
		North Carolina	15	155,709
		South Carolina	3	370,733
		Virginia	15	131,439
		West Virginia	1	392,351
	**East South Central**		15	288,463
		Alabama	5	223,458
		Kentucky	1	996,488
		Mississippi	1	694,045
		Tennessee	8	189,891
	**West South Central**		31	298,733
		Arkansas	3	222,577
		Louisiana	3	363,228
		Oklahoma	4	220,527
		Texas	21	315,296
**West**			262	67,883
	**Mountain**		55	97,547
		Arizona	8	190,750
		Colorado	21	60,902
		Idaho	4	91,376
		Montana	5	43,161
		Nevada	8	83,522
		New Mexico	5	91,243
		Utah	2	363,970
		Wyoming	2	63,250
	**Pacific**		207	60,002
		Alaska	6	27,969
		California	152	61,740
		Hawaii	3	103,715
		Oregon	12	75,968
		Washington	34	48,391

**Table 2:** Types of services offered by abortion care facilities (N=780) by US region and state, May 2017.

Region and state			Facilities offering only aspiration or surgical abortion, n (%)	Facilities offering only medication abortion, n (%)	Facilities offering both aspiration or surgical abortion and medication abortion, n (%)	Latest gestational limit (weeks since LMP^a^) as listed on website
United States			34 (4.4)	236 (30.3)	510 (65.4)	28
**Northeast**			18 (7.7)	89 (38.2)	126 (54.1)	27
	**New England**		4 (5.4)	34 (45.9)	36 (48.6)	27
		Connecticut	0 (0)	13 (65.0)	7 (35.0)	<24
		Maine	0 (0)	17 (85.0)	3 (15.0)	<19
		Massachusetts	4 (21.1)	0 (0)	15 (78.9)	27
		New Hampshire	0 (0)	1 (16.7)	5 (83.3)	<16
		Rhode Island	0 (0)	0 (0)	3 (100)	<22
		Vermont	0 (0)	3 (50.0)	3 (50.0)	<19
	**Middle Atlantic**		14 (8.8)	55 (34.6)	90 (56.6)	<25
		New Jersey	6 (12.0)	22 (44.0)	22 (44.0)	<25
		New York	8 (8.7)	29 (31.5)	55 (59.8)	24
		Pennsylvania	0 (0)	4 (23.5)	13 (76.5)	23
**Midwest**			2 (2.2)	23 (25.0)	67 (72.8)	24
	**East North Central**		1 (1.5)	16 (23.5)	51 (75.0)	24
		Indiana	0 (0)	1 (16.7)	5 (83.3)	<14
		Illinois	0 (0)	9 (36.0)	16 (64.0)	24
		Michigan	0 (0)	5 (21.7)	18 (78.3)	24
		Ohio	1 (9.1)	1 (9.1)	9 (81.8)	<22
		Wisconsin	0 (0)	0 (0)	3 (100)	<23
	**West North Central**		1 (4.2)	7 (29.2)	16 (66.7)	22
		Iowa	0 (0)	6 (66.7)	3 (33.3)	<22
		Kansas	0 (0)	1 (25.0)	3 (75.0)	<22
		Minnesota	1 (20.0)	0 (0)	4 (80.0)	22
		Missouri	0 (0)	0 (0)	1 (100)	<22
		Nebraska	0 (0)	0 (0)	3 (100)	<22^b^
		North Dakota	0 (0)	0 (0)	1 (100)	16
		South Dakota	0 (0)	0 (0)	1 (100)	<14
**South**			4 (2.1)	20 (10.4)	169 (87.6)	26
	**South Atlantic**		2 (1.4)	15 (10.2)	130 (88.4)	26
		Delaware	0 (0)	1 (33.3)	2 (66.7)	<16
		District of Columbia	1 (33.3)	0 (0)	2 (66.7)	26
		Florida	1 (1.5)	5 (7.7)	59 (90.8)	24
		Georgia	0 (0)	4 (23.5)	13 (76.5)	24^c^
		Maryland	0 (0)	4 (16.0)	21 (84.0)	26
		North Carolina	0 (0)	0 (0)	15 (100)	<21^b^
		South Carolina	0 (0)	0 (0)	3 (100)	14
		Virginia	0 (0)	1 (6.7)	14 (93.3)	21
		West Virginia	0 (0)	0 (0)	1 (100)	16
	**East South Central**		1 (6.7)	2 (13.3)	12 (80.0)	22^b,c^
		Alabama	1 (20.0)	0 (0)	4 (80.0)	21
		Kentucky	0 (0)	0 (0)	1 (100)	22^b,c^
		Mississippi	0 (0)	0 (0)	1 (100)	16
		Tennessee	0 (0)	2 (25.0)	6 (75.0)	<18
	**West South Central**		1 (3.2)	3 (9.7)	27 (87.1)	<24^d^
		Arkansas	0 (0)	2 (66.7)	1 (33.3)	21
		Louisiana	0 (0)	0 (0)	3 (100)	<19^b,c^
		Oklahoma	0 (0)	1 (25.0)	3 (75.0)	<22^b^
		Texas	1 (4.8)	0 (0)	20 (95.2)	<24^d^
**West**			10 (3.8)	104 (39.7)	148 (56.5)	28
	**Mountain**		4 (7.3)	17 (30.9)	34 (61.8)	28
		Arizona	0 (0)	1 (12.5)	7 (87.5)	24
		Colorado	2 (9.5)	8 (38.1)	11 (52.4)	26
		Idaho	0 (0)	2 (50.0)	2 (50.0)	<16
		Montana	0 (0)	2 (40.0)	3 (60.0)	21
		Nevada	2 (25.0)	2 (25.0)	4 (50.0)	24
		New Mexico	0 (0)	1 (20.0)	4 (80.0)	28
		Utah	0 (0)	0 (0)	2 (100)	<22
		Wyoming	0 (0)	1 (50.0)	1 (50.0)	12^b^
	**Pacific**		6 (2.9)	87 (42.0)	114 (55.1)	26
		Alaska	0 (0)	1 (16.7)	5 (83.3)	<14
		California	5 (3.3)	67 (44.1)	80 (52.6)	24
		Hawaii	0 (0)	0 (0)	3 (100)	<24^b^
		Oregon	0 (0)	4 (33.3)	8 (66.7)	23
		Washington	1 (2.9)	15 (44.1)	18 (52.9)	26

^a^LMP: last menstrual period.

^b^Information on highest gestational limit obtained through phone call to facility.

^c^Information obtained in early 2017, and as of September 22, 2017, may no longer be accurate as a result of state laws or staffing changes.

^d^One facility in Texas listed the gestational limit on their website as <24 weeks; this was likely an error, as at the time of data collection, Texas had restrictions on abortion services after 22 weeks.

### Abortion Deserts

**Figure 1:**
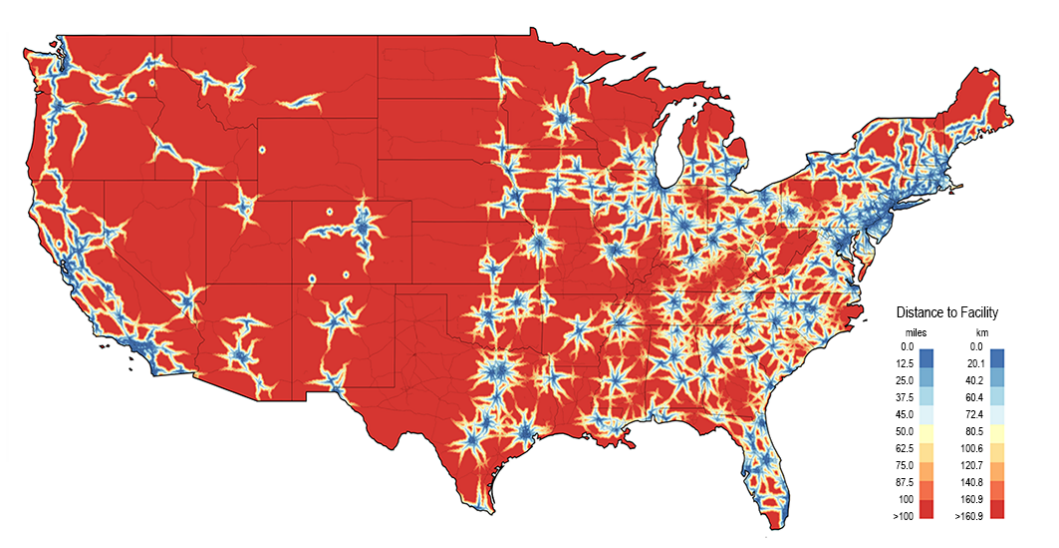
Distance to nearest abortion facility in the contiguous United States, May 2017.

We identified 27 “abortion deserts,” cities from which people would have to travel over 100 miles (160 km) to reach an abortion facility (Table 3). People living in Rapid City, SD had to travel the farthest, 318 miles (512 km), to reach an abortion facility. Although the most cities in any one state (n=10) were located in Texas, there was a wide geographic diversity, with 15 unique states represented. These states were overwhelmingly in the South and Midwest. Figure 1 shows the geographic distribution of these distances, where large areas of the Midwest and Southwest had no abortion facility.

**Table 3:** Abortion deserts (cities >100 miles/160 km to closest facility) in the United States, May 2017.

City and state		2015 population, n	Distance to closest facility, miles (km)	Location of closest facility
1	Rapid City, SD	73,569	318 (512)	Billings, MT
2	Lubbock, TX	249,042	308 (496)	Fort Worth, TX
3	Midland, TX	132,950	293 (472)	Fort Worth, TX
4	Odessa, TX	118,968	282 (454)	El Paso, TX
5	Amarillo, TX	198,645	258 (415)	Warr Acres, OK
6	Casper, WY	60,285	223 (359)	Fort Collins, CO
7	San Angelo, TX	100,450	204 (328)	Austin, TX
8	Bismarck, ND	71,167	196 (315)	Fargo, ND
9	Laredo, TX	255,473	160 (257)	San Antonio, TX
10	Lake Havasu City, AZ	53,553	144 (232)	Henderson, NV
11	Abilene, TX	121,721	144 (232)	Fort Worth, TX
12	Corpus Christi, TX	324,074	139 (224	San Antonio, TX
13	La Crosse, WI	52,306	137 (220)	Madison, WI
14	Springfield, MO	166,810	136 (219)	Fayetteville, AR
15	Lake Charles, LA	76,070	132 (212)	Baton Rouge, LA
16	Wichita Falls, TX	104,710	123 (198)	Fort Worth, TX
17	Columbia, MO	119,108	122 (196)	St Louis, MO
18	Evansville, IN	119,943	122 (196)	Bloomington, IN
19	St George, UT	80,202	121 (195)	Las Vegas, NV
20	Manhattan, KS	56,308	120 (193)	Overland Park, KS
21	Green Bay, WI	105,207	119 (192)	Milwaukee, WI
22	Pocatello, ID	54,441	114 (183)	Twin Falls, ID
23	Victoria, TX	67,574	107 (172)	San Antonio, TX
24	Fort Wayne, IN	260,326	106 (171)	Toledo, OH
25	Owensboro, KY	59,042	105 (169)	Louisville, KY
26	Dothan, AL	68,567	103 (166)	Montgomery, AL
27	Chattanooga, TN	176,588	101 (163)	Marietta, GA

## Discussion

### Principal Results

Using an online search method, we identified almost 800 abortion facilities in the United States, which is consistent with other estimates of abortion clinics and nonspecialized clinics providing abortion [3]. These facilities were not distributed proportionately by state population. Through our analysis, we also found that 27 US cities, largely in the Midwest and the South, could be characterized as abortion deserts, as they did not have a publicly advertised abortion facility within 100 miles (160 km). These findings are consistent with those published by Bearak and colleagues [6], who found that the US counties where women would have to travel the farthest to reach the nearest abortion clinic were concentrated in the middle of the country, as well as several metropolitan areas in Texas. The lack of access to a common reproductive health service such as abortion is a public health concern in that more women in these cities could be forced to carry unwanted pregnancies to term if they are unable to travel long distances to obtain abortion care.

As states continue to pass, implement, and defend restrictions on abortion [8], it is possible that the number of abortion facilities will continue to decrease in those states with the most restrictions. The 6 states that have only 1 abortion facility have combined populations of almost 4 million women of reproductive age who will be forced to travel out of their home state to access abortion care if those facilities close.

For people seeking abortion services in the cities characterized as abortion deserts and in states with few facilities, reaching a facility for care could be incredibly challenging. Access to transportation is a barrier for people seeking all types of health care, in both urban and rural settings [28]. Lower-income women who are unable to access a car or money for gas may have to travel by bus, train, or other forms of transportation, which also becomes more difficult the farther they have to travel. Delays in care due to distance or transportation can push women seeking abortion to later gestations [16,29,30] and are likely to disproportionately affect low-income women, who may struggle to cover the cost of transport [11,14]. Delays to abortion care may be particularly crucial to women in Wyoming, Alaska, Indiana, South Dakota, and South Carolina, where the abortion facilities had the lowest gestational limits. We found that 26.5% of identified facilities performed abortions at 20 weeks or later, which is lower than estimates from 2011-2012 [31], perhaps due to an increased number of state restrictions on abortion after 20 weeks since those estimates were published.

It seems likely that the larger number of facilities in the Northeast and West can be attributed to the fact that 40% to 50% of identified facilities in those regions are offering medication abortion only. The high proportion of facilities offering only medication abortion reflects the opportunities provided by medication abortion: the skills required for clinicians to provide it are minimal (compared with aspiration or surgical abortion) and the large majority of abortions in the United States (80.5%) occur at or before 10 weeks’ gestation (the current accepted limit by which medication abortion can be provided) [32]. While the proportion of women choosing this method of abortion now accounts for 31% of nonhospital abortions (compared with 6% in 2001) [3], it is difficult to determine what the true demand would be if both medication and aspiration abortion were equally available. However, in states such as California, where fewer barriers to access exist for both types of abortion, medication abortion is now up to 46% of abortions in some populations, such as Medicaid recipients [33]. Additionally, states in the Northeast and West are less likely to have laws that limit the provision of medication abortion to physicians [34] and more likely to have policies that allow nurse practitioners, certified nurse midwives, and physicians assistants to offer medication abortion as part of their scope of practice.

These findings underscore the opportunities to pursue geographic expansion and other innovative models to achieve more equitable access to abortion care [35]. Some states have already managed this through an expansion of medication abortion-only services and increased use of telemedicine, which has been demonstrated to be safe and acceptable to women and to decrease travel for patients [36]. Indeed, in this analysis, Maine had the lowest ratio of women of reproductive age per facility, which was likely the result of an expansion of medication abortion through telemedicine programs offered from the existing Maine facilities [37]. While 19 states (almost exclusively in the South and Midwest) effectively prohibit telemedicine medication abortion [34], a recent Iowa Supreme Court decision could have implications for other states challenging similar restrictions that would allow expansion of medication abortion provision [38]. Given that it is less resource intensive, existing health care providers in the Midwest and the South, particularly in states where there is only 1 abortion provider or those states that contain cities classified as abortion deserts, could consider filling gaps in access by offering medication abortion alone as an entry point into abortion care, especially for primary care providers. Expanding the types of providers who can offer aspiration and medication abortion, such as nurse practitioners, would also increase the number of providers in smaller urban areas, thus expanding access to care [39]. However, it is important to note that in some states in which half or more facilities are only providing medication abortion, such as Idaho and Wyoming, the other facilities in the state offer abortion care up to 16 weeks and 12 weeks, respectively. Simply increasing the availability of medication abortion would not meet the needs of all women seeking abortion, some of whom may prefer aspiration abortion or need later abortion.

Supportive policy related to transportation for reproductive health services could also help reduce the burden on women in abortion deserts who have to travel extended distances to access services. California has recently introduced a Medicaid benefit to provide transportation for reproductive health care services (including abortion) to enrollees [40]. Further research is needed to determine what other policies can be enacted to reduce burdens of transportation and distance.

The internet will likely continue to be a key place for people to obtain the locations of abortion-providing facilities. However, both reduced geographic access and a desire to have more privacy and autonomy around the abortion process may lead women to seek out information on self-abortion [18] and obtain medication abortion pills through online sources, many of which have recently been shown to be selling effective medications with delivery to US-based mailing addresses [41]. There are no accurate estimates of how many women are obtaining abortion pills online, but the existence of online marketplaces and the documented feasibility of ordering from them implies online purchasing is occurring at volume.

### Strengths and Limitations

This study is unique in that it systematically documented what people searching for abortion services online would find in search engine listings in early 2017 from a patient-centered perspective. A strength of this study is that it used up-to-date information on facilities of any volume to calculate distances, while other recent studies have been limited to a 2014 iteration of a proprietary database of only high-volume abortion-providing facilities (>400 abortions per year) that the Guttmacher Institute maintains [3,6]. An additional strength of the study is that it did not include abortion providers that offer abortion only to their existing patients or those that do not advertise their services, which would distort an accurate portrayal of the visibility of abortion availability. In addition, this analysis included the maximum gestations at which abortions were provided in each state, regardless of the state laws.

However, this study also has limitations. We used search terms that would enable us to locate abortion facilities in specific cities and states, but someone seeking abortion care might search “abortion clinic near me” and their results could vary from ours based on the location they are searching from. We attempted to eliminate geolocation bias by searching in Incognito mode and clearing both cache and cookies after each search. The information provided here is limited to what women seeking services would encounter—information that facilities chose to make available on their websites and provided through mystery shopper calls. Website information may be inaccurate or updated infrequently. It is possible that, if a woman called a facility to describe her unique situation, the staff could provide her with information about additional services that they do not wish to list on their website.

Distance is not the only barrier that people may face in trying to access abortion services—they may also face abortion stigma, waiting periods [13], and state gestational limits [16,42] as a result of state-level restrictions. In addition, the abortion facility that is closest to where a woman lives may not meet all her needs, particularly if it only provides medication abortion, has low limits on the gestation at which it provides abortion care, or cannot serve women with health conditions who may have higher risks.

### Conclusions

Online searches provide information about abortion facilities and their services. The locations of these providers are not distributed equitably geographically across the United States. Having to travel long distances for abortion care can disproportionately affect low-income women and potentially push women to seek abortion at later gestations when care is even less available. Travel burdens may exist in addition to other restrictions on abortion in the state, including waiting periods and gestational limits, which can exacerbate inequities in the ability to access abortion care as part of the full range of reproductive health services.

## Abbreviations

LMP: last menstrual period
